# Combining phylogeography and climate models to track the diversification and spread of *Phlebotomus simici*

**DOI:** 10.1038/s41598-025-94601-1

**Published:** 2025-03-25

**Authors:** Edwin Kniha, Stephan Koblmüller, Katharina Platzgummer, Oscar Kirstein, Debora Diaz, Vít Dvořák, Ozge Erisoz Kasap, Betim Xhekaj, Kurtesh Sherifi, Julia Walochnik, Attila J. Trájer

**Affiliations:** 1 Institute of Specific Prophylaxis and Tropical Medicine, Center for Pathophysiology, Infectiology and Immunology, Medical University of Vienna, Vienna, Austria; 2 Institute of Biology, University of Graz, Graz, Austria; 3 Laboratory of Entomology, Ministry of Health, Jerusalem, Israel; 4 Department of Parasitology, Faculty of Science, Charles University, Prague, Czech Republic; 5 Department of Biology, Ecology Section, Faculty of Science, VERG Laboratories, Hacettepe University, Ankara, Turkey; 6 Faculty of Agriculture and Veterinary, University of Prishtina, Prishtina, Kosovo; 7 Sustainability Solutions Research Lab, University of Pannonia, Veszprém, Hungary

**Keywords:** Central Europe, Phylogeography, *Adlerius*, Divergence time, COI, Animal migration, Infectious diseases, Biogeography, Evolutionary biology, Phylogenetics, Animal migration, Evolutionary developmental biology, Zoology, Palaeoclimate

## Abstract

Phlebotomine sand flies (Diptera: Psychodidae: Phlebotominae) are the principal vectors of *Leishmania* spp. (Kinetoplastida: Trypanosomatidae) worldwide. The subgenus *Adlerius* is taxonomically challenging and currently comprises about 20 species with a wide geographic distribution from eastern Asia to southeastern Europe. Some species are confirmed or suspected vectors of *Leishmania donovani*/*infantum*, *L. major*, and *L. tropica*, and are thus of high medical and veterinary relevance. A single record of *Phlebotomus* (*Adlerius*) *simici* in Austria from 2018 marks its sporadic northernmost and westernmost occurrence, with the origin of its appearance remaining unclear. To better understand *Adlerius* diversification and particularly post-glacial spread of *Ph. simici* to northern parts of Europe, we combined phylogenetic analyses with climatic suitability modelling. Divergence time estimates well supported the currently observed geographic distribution of the studied species and revealed several taxonomic challenges in the subgenus. We clearly delineated three distinct genetic and geographic *Ph. simici* lineages and phylogeographically assessed diversification that were well supported by climatic models. This study provides a comprehensive phylogenetic analysis of the subgenus *Adlerius*, enhancing our understanding of the diversification in relation to changing climate of this understudied group, and we present new insights into the post-glacial spread of *Ph. simici*, a suspected vector of *L. infantum*.

## Introduction

Phlebotomine sand flies (Diptera: Psychodidae: Phlebotominae) are small hematophagous insects and the principal vectors of the protozoan parasites *Leishmania*, the causative agents of leishmaniasis. They inhabit tropical, subtropical, arid, and temperate regions worldwide^1^. Species of the subgenus *Adlerius* Nitzulescu, 1931, show a wide geographic distribution range, being endemic in China, Middle Eastern countries (e.g. Iran, Iraq, Israel, Lebanon and Syria), Türkiye and Greece including Aegean islands, and many Balkan countries as far north as Serbia^2–5^.

*Adlerius* is a subgenus little studied, which presents a taxonomically interesting and challenging group. Its morphological characterization is inconclusive for some valid taxa, as females of some species are morphologically indistinguishable, for example, *Phlebotomus chinensis* and *Phlebotomus sichuanensis* in China^6^, or *Phlebotomus brevis* and *Phlebotomus simici* that have a sympatric distribution in Lebanon^7^. The last systematic revision of the subgenus, now outdated by over four decades, was based solely on the assessment of morphological characters and regarded some of them inadequate for conclusive species identification^8^. A few later studies using also molecular techniques enabled the description of a new species, *Phlebotomus creticus* on the island of Crete^9^, suggested the presence of previously undescribed *Adlerius* species from Türkiye and Armenia^4,10^, and another from Moldova^11^ and proposed resurrection of previously synonymized *Ph. davidi* as a valid species^12^. Albeit the use of molecular taxonomy approaches has been only sporadic in *Adlerius* species, the data obtained clearly highlights the need of a careful revision of the subgenus, based on a complex assessment of a combination of morphological as well as molecular markers. In addition to its taxonomical peculiarities, the subgenus *Adlerius* is also of medical and veterinary relevance as some species are proven competent vectors for several *Leishmania* species, such as *Ph. chinensis* for *L. infantum*/*donovani* and *Ph. arabicus* for *L. tropica*^13,14^, or suspected vectors such as *Ph. halepensis* for *L. major* and *L. tropica*, or *Ph. simici* and *Ph. balcanicus* for *L. infantum*^15–17^. Their proven or expected involvement in various transmission cycles of *Leishmania* spp. suggests that other species of the subgenus, whose vectorial competence has never been tested, may also be relevant for understanding the epidemiology of sand fly-borne diseases in the regions of their presence.

In Europe, *Phlebotomus mascittii* is the most widespread species and in some countries north of the usual sand fly Mediterranean distribution, it is the only endemic species^18^. Unexpectedly, a single female *Ph. simici* specimen was recorded in Austria in 2018, marking the western- and northernmost occurrence of an *Adlerius* species^10^. Despite many trapping efforts since then, it has never been collected again in Austria, and neither in any bordering countries. Kniha et al.^10^ identified three distinct lineages of *Ph. simici* from Israel, Türkiye and the Balkans, respectively, suggesting a natural or anthropogenic post-glacial dispersal from a Balkan country to Austria.

Several previous studies showed that the western branch of the Paratethys and the Aegean-Asia Minor region could have played an important role in the diversification of Eastern Mediterranean sand fly taxa^19,20^. The evolutionary history of phlebotomine sand flies, particularly within the subgenera *Adlerius*, *Paraphlebotomus*, and *Transphlebotomus*, reflects a complex interplay of climatic, geographic, and ecological transformations across the Oligocene, Miocene, and Quaternary periods. Key geological and climatic shifts in the peri-Mediterranean region shaped both the distribution and diversification of sand fly species, influencing their speciation, range expansion, and ecological adaptation. During the Oligocene and early Miocene epochs, the humid, subtropical climate of the Mediterranean basin posed limited suitability for many sand fly ancestors, particularly for those with Mediterranean affinities^21,22^. However, with the gradual transition to a drier subtropical climate by the middle Miocene, habitats became more conducive to the diversification of certain sand fly groups, including *Larroussius* and *Paraphlebotomus*. Modelling studies of climate suitability from the Rupelian to the Tortonian stages in the Central Paratethys suggest that the ancestor of the *Larroussius* group could have adapted well to these evolving conditions, while the Central Paratethys may have served as a geographic barrier to eastward-migrating ancestors of *Paraphlebotomus* and *Phlebotomus* species^22^. As the Miocene progressed, these barriers began to fragment with the tectonic subsidence of landmasses like the Hellene Orogenic Belt, facilitating niche expansion and the gradual migration of *Phlebotomus* species, which adapted to the more extreme Mediterranean climate^23^.

The diversification of various sand fly species is linked to significant paleogeographic and paleoclimatic events, particularly the desiccation of the Mediterranean Basin during the Messinian Salinity Crisis^24,25^. This event led to the isolation and subsequent divergence of sand fly populations, driving the initial stages of speciation^20^. Following the Zanclean re-flood of the Mediterranean Basin, *Phlebotomus* species began to reoccupy new habitats, with many adapting to the distinct climatic regimes of the Iberian, Apennine, and Balkan refugia^26^. These refugia offered stable microclimates through glacial maxima, fostering the differentiation of sand flies into “Trans-Mediterranean,” “East Mediterranean,” and “West Mediterranean” climatic groups^27^.

The *Adlerius* group encapsulates the effects of both ancient and recent climatic forces, and thus can provide understanding of Mediterranean sand fly evolution^19,20^. While phylogeographic reconstructions of the region highlight the role of the Mediterranean Sea and adjacent landforms in shaping the evolutionary pathways of sand flies, the genetic, ecological, and bioclimatic adaptations of *Adlerius* underscore the adaptability of sand flies to shifting ecological landscapes. As vectors of *Leishmania*, their evolutionary history offers crucial insights into the biogeographical and ecological processes that continue to influence disease transmission dynamics in these regions, shedding light on both ancient speciation events and contemporary distributions.

The present study aimed to assess phylogenetic relationships of *Adlerius* species and reconstruct precise temporal patterns of diversification of *Ph. simici* lineages by combining phylogeographic data and climate modelling. Results should further elucidate and support our understanding of the post-glacial dispersal and the currently known distribution.

## Results

### Phylogenetic relationships of *Adlerius*

**Fig. 1:**
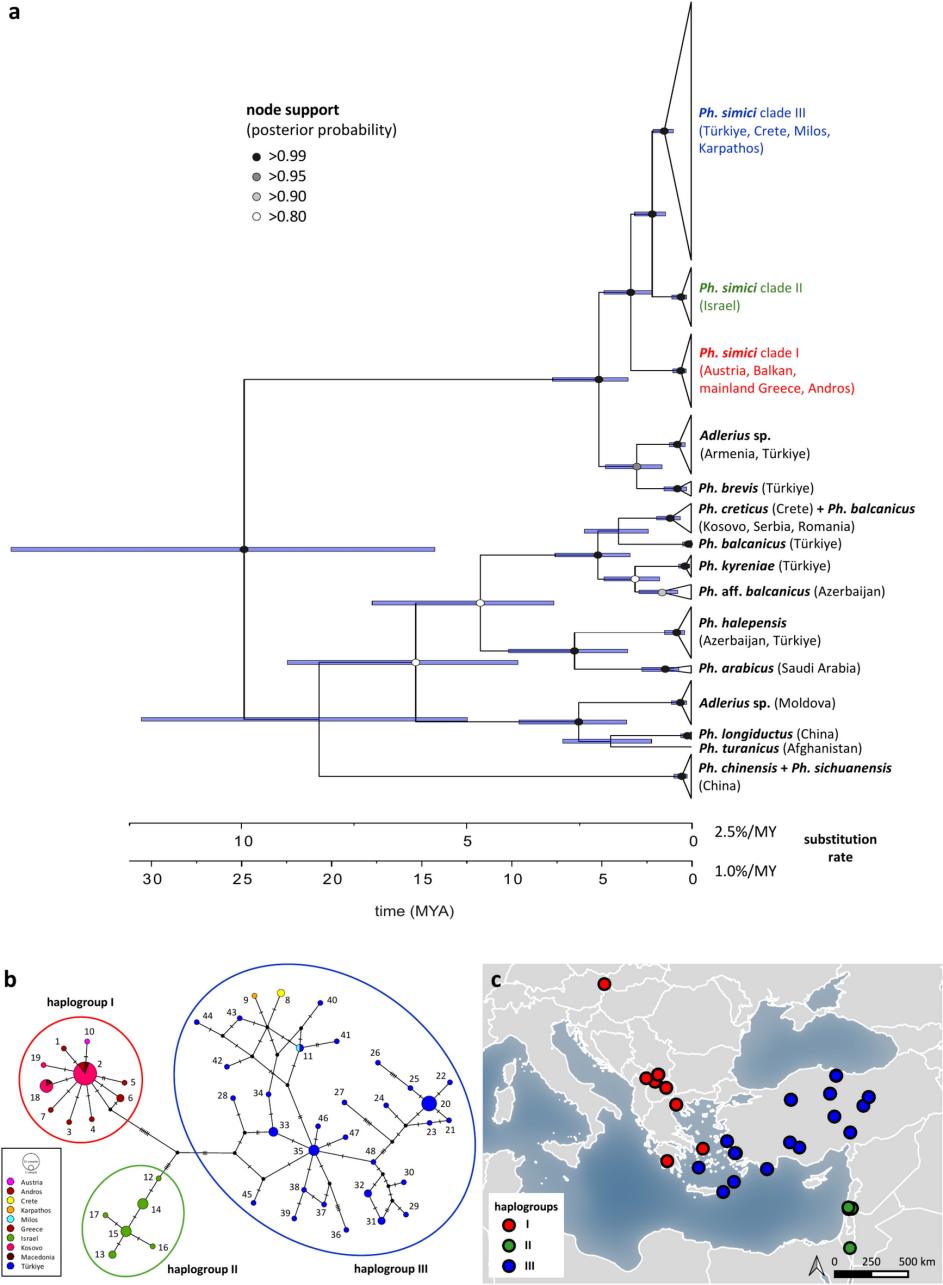
Dated phylogeny of *Adlerius* based on COI sequences and corresponding *Ph. simici* haplotypes. Timetree with divergence times calculated assuming a maximum and minimum substitution rate of 2.5% and 1.0% per million years (my) for COI, respectively (**a**). The tree shows the median divergence ages, with the node bars representing the 95% highest posterior density (HPD) intervals for node ages. Node support is only shown for those nodes with posterior probabilities > 0.8. Statistical parsimony network of *Phlebotomus simici* from six countries (including four Greek islands) based on COI sequences (**b**). Colored circles represent major haplogroups identified. Geographical origin of identified *Ph. simici* haplogroups (I–III) among analyzed samples (**c**). Macedonia = North Macedonia.

Phylogenetic analyses based on COI sequences revealed two major clades: one comprising *Ph. simici*, *Ph. brevis* and an unidentified *Adlerius* species from Armenia and Türkiye (Fig. 1a), the second, major clade comprising all other included *Adlerius* species (Fig. 1a). In the first major clade, *Ph. simici* was observed to form three distinct lineages. *Phlebotomus simici* lineage I comprised specimens from Austria, Balkan, Greece and Andros, lineage II all specimens from Israel and lineage III all specimens from Türkiye, Crete, Milos and Karpathos. Lineages II and III were resolved as sister groups, together forming the sister group of *Ph. simici* lineage I. *Phlebotomus brevis* and *Adlerius* sp. resulted as sister species, together forming the sister group of *Ph. simici* (Fig. 1a).

In the second major clade, *Phlebotomus balcanicus* (incl. *Ph.* aff*. balcanicus*) comprised three different groups. *Phlebotomus balcanicus* originating from Kosovo, Serbia and Romania constituted the first clade together with the closely related species *Ph. creticus*, forming the sister group of *Ph. balcanicus* from Türkiye (Fig. 1a). *Phlebotomus* aff. *balcanicus* from Azerbaijan formed the sister group of *Ph. kyreniae* from Türkiye and together represented the sister group of *Ph. balcanicus* (Balkan)/*Ph. creticus* + *Ph. balcanicus* (Türkiye). Further, *Ph. halepensis* and *Ph. arabicus* formed sister species, and *Ph. longiductus* + *Ph. turanicus* were resolved as the sister group of an unidentified *Adlerius* species from Moldova. *Phlebotomus chinensis* and *Ph. sichuanensis*, which cannot be discriminated by COI sequences, resulted as a rather distinct lineage (Fig. 1a).

### Divergence times estimates

Median divergence time estimates of all included *Adlerius* species ranged from 25 million years ago (mya) (based on 1.0% divergence rate/my) and 10 mya (based on 2.5% divergence rate/my) to 2.5 mya and 1 mya. The majority of major splits were estimated to be between 6.5 mya (1.0% divergence/my) and 1 mya (2.5% divergence/my). Within major clade 1, the split of *Ph. simici* from *Ph. brevis* + *Adlerius* sp. were estimated to have happened between 5 mya and 2.3 mya. The first split between *Ph. simici* lineage I and lineage II + lineage III appeared between 3.5 mya and 1.25 mya, and the split between *Ph. simici* lineage II and lineage III was estimated between 2.5 mya and 1 mya (Fig. 1a).

Divergence times estimates involving *Ph. balcanicus* in the second major clade were similar to divergence times observed in the first major clade. The first split between *Ph. creticus*/*Ph. balcanicus* (Balkan) + *Ph. balcanicus* (Türkiye) and *Ph. kyreniae* + *Ph.* aff. *balcanicus* appeared between 5 mya and 2.3 mya, the split between *Ph. creticus*/*Ph. balcanicus* (Balkan) and *Ph. balcanicus* (Türkiye) was estimated between 4 and 2 mya. Finally, the split between *Ph. kyreniae* and *Ph.* aff. *balcanicus* was estimated between 3 mya and 1.1 mya (Fig. 1a). Also, divergence time estimates between 6.5 mya and 2.5 mya were obtained for *Ph. halepensis* and *Ph. arabicus* as well as *Ph. longiductus* + *Ph. turanicus* and the unidentified *Adlerius* species from Moldova (Fig. 1a).

### Phylogeographic patterns of *Ph. simici*

Overall, 48 COI haplotypes of *Ph. simici* were identified, comprising 49 variable sites (29 parsimony informative) with a haplotype diversity (Hd) of 0.946, a nucleotide diversity (π) of 0.0195, and a mean group distance of 1.9% (S.E. = 0.4%). Three major haplogroups (I–III) corresponding to the three *Ph. simici* lineages of the phylogenetic tree were observed (Fig. 1b), revealing a distinct geographic origin (Fig. 1c). Haplogroup I (pd = 0.2%, S.E. = 0.07%) comprised specimens from Austria, Kosovo, North Macedonia, mainland Greece and the Aegean Greek island Andros. Haplogroup II (pd = 0.3%, S.E. = 0.1%) comprised all specimens originating from Israel, and haplogroup III (pd = 1.3%, S.E. = 0.3%) included all specimens from Türkiye as well as the Aegean Greek islands of Crete, Milos and Karpathos (Fig. 1b). The mean group distance between haplogroup I and II was 2.6% (S.E. = 0.7%), between haplogroup I and III was 2.6% (S.E. = 0.7%), and 2.3% (S.E. = 0.6%) between haplogroup II and III (Supplementary Table 1).

**Fig. 2:**
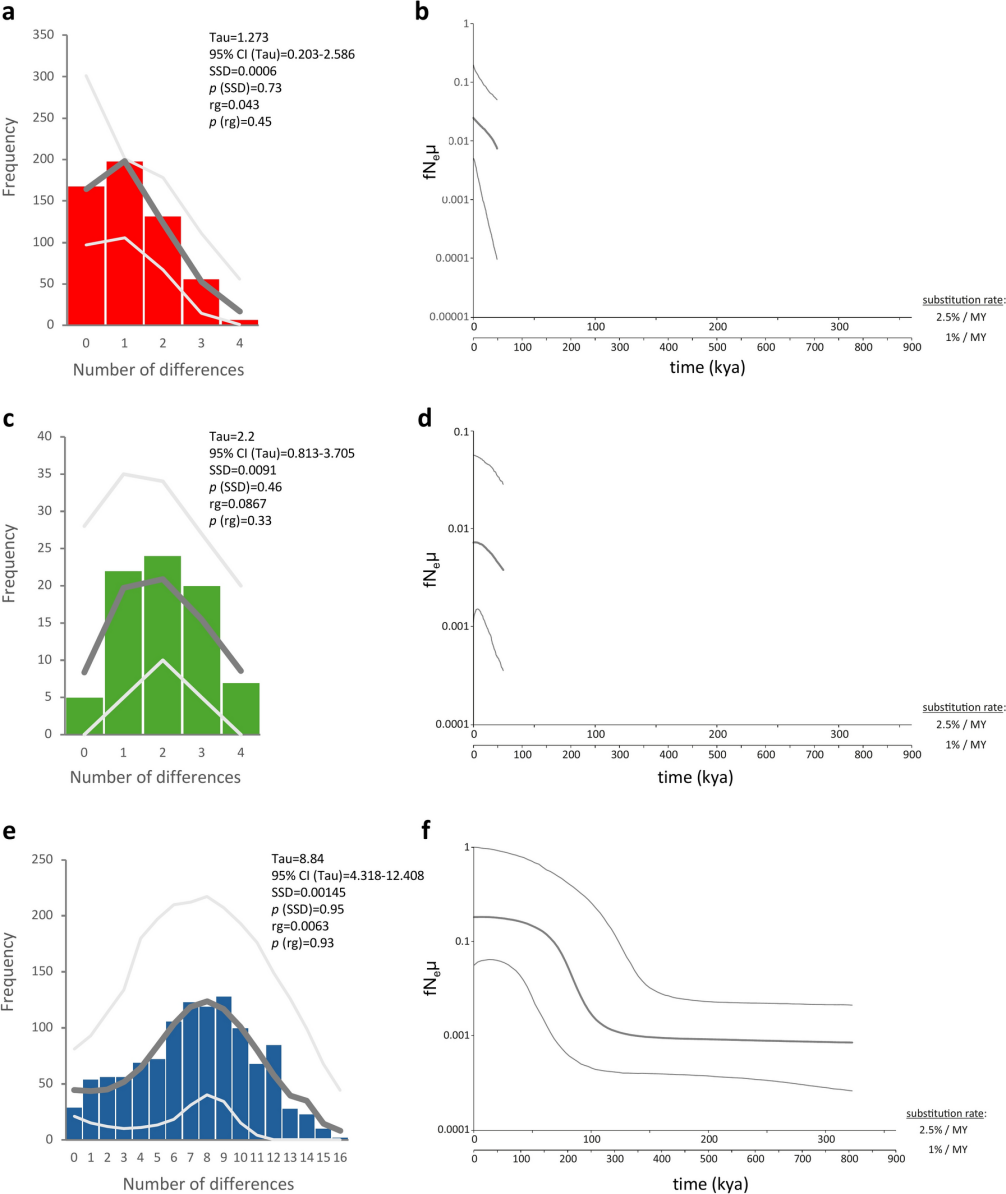
Signatures of population expansion in *Phlebotomus simici*. Mismatch distributions for *Ph. simici* haplogroup I (**a**), haplogroup II (**c**), and haplogroup III (**e**), based on COI sequences. Black columns represent the observed frequency of pairwise differences. Gray lines refer to the expected distribution based on parameter estimates and their 95% confidence limits simulated under a model of population growth. The sum of squared differences (SSD) and raggedness index (rg) and their respective P values are given to describe the fit of the observed mismatch distribution to the expectation based on growth parameter estimates. Estimated Bayesian Skyline Plots (BSPs) for all three haplogroups (**b**, **d**, **f**), assuming a minimum and maximum substitution rate for mitochondrial protein-coding genes of insects of 1% and 2.5% per my. The thick line denotes the median estimate; thin lines indicate the 95% highest posterior density (HPD) interval. fNeµ = female effective population size scaled by substitution rate.

For the star-shaped haplogroup I, a significantly negative Tajima’s D (D = − 2.235; p = 0.0.002), and Fu’s Fs (Fs = − 28.866; p < 0.001), and a unimodal mismatch distribution (Fig. 2a) with non-significant raggedness index (rg = 0.043; p = 0.447) and non-significant sum of squared differences (SSD = 0.001; p = 0.726) 0.0867; p = 0.33), indicated a recent population expansion. The Bayesian Skyline plot clearly demonstrated evidence of a strong, recent, post-glacial population expansion (Fig. 2b). The time to the most recent common ancestor was estimated at 54.0 (95% HPD interval 20.7–101.1) kya to 135.1 (95% HPD interval 51.8–252.9) kya, depending on the assumed substitution rates. For haplogroup II, a negative, but non-significant, Tajima’s D (D = − 0.387; p = 0.395), a significantly negative Fu’s Fs (Fs = − 16.072; p < 0.001), and a unimodal mismatch distribution (Fig. 2c) with non-significant raggedness index (rg = 0.087; p = 0.328) and sum of squared differences (SSD = 0.009; p = 0.461), indicated a recent population expansion. Also, the Bayesian Skyline plot provided clear evidence for strong recent post-glacial population expansion (Fig. 2d). The time to the most recent common ancestor was estimated at 81.9 (95% HPD interval 24.6–153.3) kya to 204.7 (95% HPD interval 61.4–383.3) kya, depending on the assumed substitution rate. For haplogroup III, a negative, but non-significant, Tajima’s D (D = − 0.794; p = 0.237), a significantly negative Fu’s Fs (Fs = − 25.012; p < 0.001), and a skewed unimodal mismatch distribution (Fig. 2e) with non-significant raggedness index (rg = 0.006; p = 0.928) and non- significant sum of squared differences (SSD = 0.001; p = 0.945), indicated past population expansion. The Bayesian Skyline plot provided clear evidence for population expansion, but earlier than the two other haplogroups (Fig. 2f). The time to the most recent common ancestor was estimated at 561.6 (95% HPD interval 325.1–807.2) kya to 1403.9 (95% HPD interval 812.8–2018.1) kya, depending on the assumed substitution rate.

**Fig. 3:**
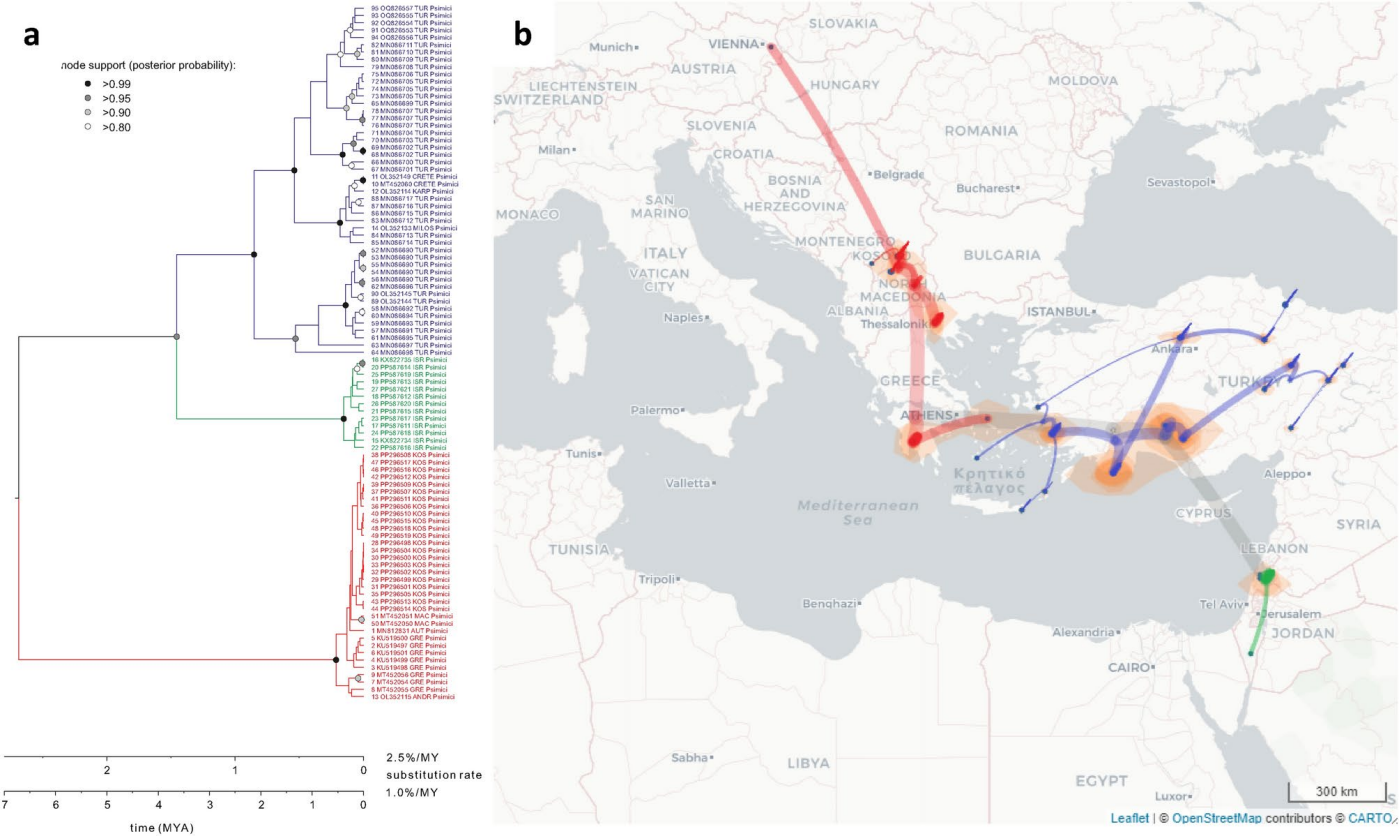
Phylogeographic scenario of the spread of *Ph. simici* based on COI sequences. (**a**) Intraspecific time-calibrated MCC tree of *Ph. simici*, assuming a minimum and maximum substitution rate of 1% and 2.5% per my for the COI gene in insects. The tree shows the median divergence ages; node support is only shown for nodes with posterior probabilities > 0.8. Different colors indicate to the distinct *Ph. simici* main clades. (**b**) Map showing the transition paths of the spread of *Ph. simici*. Transition paths are colored according to clades in (**a**); width and opacity of transition paths reflect their depth of the transition in the phylogenetic tree (older paths are wider and more transparent). Orange areas indicate 80% HPD intervals for ancestral locations. The figure was created with EvoLaps2 v.2.42 (https://www.evolaps.org).

Our spatial diffusion analysis suggested that the most recent common ancestor (MRCA) of all extant *Ph. simici* (or more specifically, their mtDNA) occurred in southwestern Türkiye. From this region, the species expanded its range westward to Greece and further into the Balkans, reaching as far north as Austria (lineage I), and eastward, where it subsequently colonized the Near East (Israel; clade II) and spread further within Türkiye, including the southern Aegean islands (lineage III). In clade I (MRCA ~ 200–500 kya), the initial divergence occurred between southern Greece and the rest of the Balkans. Notably, the divergence of the Austrian sample from the central Balkan samples was estimated to have occurred 60–150 kya, depending on the substitution rate used. Lineage II was exclusively found in Israel, with levels of intra-clade divergence comparable to those observed in clade I. The MRCA of lineage III was inferred to have occurred in central southern Türkiye, from where it expanded through Türkiye via three distinct routes. The first lineage, which colonized central Türkiye, branched off approximately 0.8–2.0 mya. Initially present in southern central Türkiye near the Mediterranean coast (with the MRCA dating to ~ 0.5–1.25 mya), it spread toward the north and east more recently. The split between the other two major lineages within clade III occurred approximately 0.5–1.25 mya in southwestern Türkiye. One of these lineages, with an MRCA dating to ~ 300–750 kya, colonized the southwestern Turkish coast and progressively expanded northward, eventually reaching the central northern part of Türkiye near the Black Sea coast and also spreading to the western coast of Türkiye. The second lineage, with an MRCA of approximately 200–500 kya, colonized the western Turkish Mediterranean coast and subsequently spread to the southern Greek islands (Fig. 3).

### Elevation and climatic characterization of *Phlebotomus simici* sites

The studied *Ph. simici*-related sites were found between sea level and 1656 m a.s.l. with a mean of 387 m (SD = 388 m); 72.3% of the studied *Ph. simici* sites were found in hot-summer Mediterranean (Köppen–Geiger Csa) climate areas, followed by cold semi-arid areas (Köppen–Geiger BSk) (14.3%). An additional 8.2% of the sites were found in Mediterranean-influenced and non-Mediterranean-influenced humid continental (Köppen–Geiger Dsa, DSb, Dfb) climate regions, and 4.8% in temperate oceanic and humid subtropical (Köppen–Geiger Cfa, Cfb) climate regions. A single site (0.4%) occurred in an arid (Köppen–Geiger BWh) climate area (Supplementary Fig. 1).

Conclusively, *Ph. simici* is a sand fly species of predominantly hot summer Mediterranean and cold semi-arid (continental steppe and forest-steppe) climate regions. The dominance of the Mediterranean climate-influence is even more conspicuous when adding hot summer Mediterranean and Mediterranean-influenced humid continental climate locations, which together represent 74.6% of *Ph. simici*-inhabited sites.

### Climatic suitability patterns

#### Early late Miocene conditions

The Tortonian (11.608–7.246 mya) climatic suitability patterns of a *Ph. simici*-ancestor species with climatic limits similar to the present-day *Ph. simici* showed that climate of the large regions of Southeast Europe and the eastern part of the Mediterranean Basin was climatically suitable. These suitable areas included the southeastern part of central Europe, long coastal areas of the Pannonian Lake, the Balkan Peninsula except for the Dinarides and certain Aegean Lowland regions, Crete, the Bulgarian Lowland and ranges, large regions of Asia Minor, certain Levantine regions of the Middle East and the present-day Northern Cyrenaica region of Libya in North Africa (Supplementary Fig. 2).

#### Middle-Pliocene conditions

**Fig. 4:**
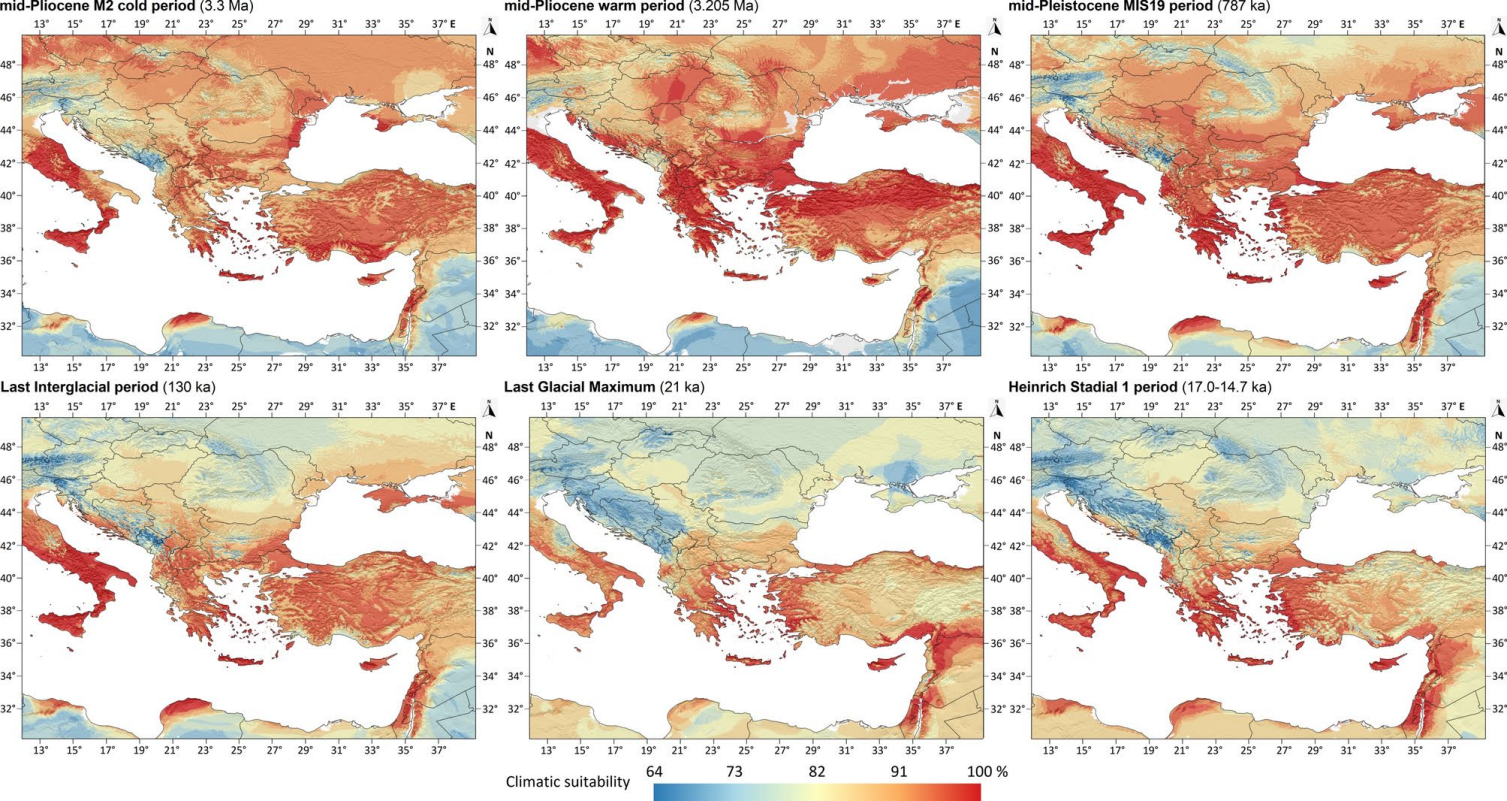
The climatic suitability patterns of *Ph. simci* in Southeast Europe and the Eastern Part of the Mediterranean Basin from the Piacenzian to the Heinrich Stadial 1. Ancient shorelines were not depicted on the maps.

In the M2 cold period of the Piacenzian (3.3 mya), the potential high climatic suitability areas of a *Ph. simici* could have been restricted to the southern regions of Asia Minor, Cyprus, the Aegean Archipelago, the Levant, the Crimean Peninsula and to the present-day western Romania in Southeast Europe and the Eastern Mediterranean Basin (Fig. 4a).

In contrast, in the warm period of the Piacenzian (3.205 mya), *Ph. simici* could have inhabited the large regions of the East Mediterranean basin including the area of the present-day continental Greece, the Aegean Archipelago, Crete, Cyprus, the North-central regions of Asia Minor, and certain regions of the Levant (Fig. 4b).

#### Quaternary conditions

In the MIS19 (787 kya) period, the modelled climatic suitability of *Ph. simici* was highest in the coastal regions of the Southern Balkan, including the whole Peloponnese Peninsula, in the Aegean Islands, including Crete, in the western coastal region of Asia Minor, in the Bosporus Region, in Cyprus, the Levantine region of the Middle east and in Northern Cyrenaica in North Africa (Fig. 4c). Compared to the MIS19 interglacial, in the Last Interglacial (130 kya), the high suitability regions were notably narrower but already covered southern parts of the Balkan and Asia Minor. The maximum suitability regions were restricted to the Aegean lands, Cyprus, and the Levant (Fig. 4d). In the Last Interglacial, the climatic suitability area was only restricted to the coastal regions of the Aegean Sea, including Euboea, Boeotia and Attica in the mainland Greece, Crete, the valley of river Büyük Menderes and the Adana Lowland in Asia Minor, as well Adana Lowland in Asia Minor, and the Levant. These regions together form the potential glacial refugia of *Ph. simici* (Fig. 4e). For the Heinrich Stadial 1, the extension of the high suitability regions increased in the South Balkan, the Aegean region, and the western part of Asia Minor, but somewhat decreased in the northern part of the Levant (Fig. 4f).

**Fig. 5:**
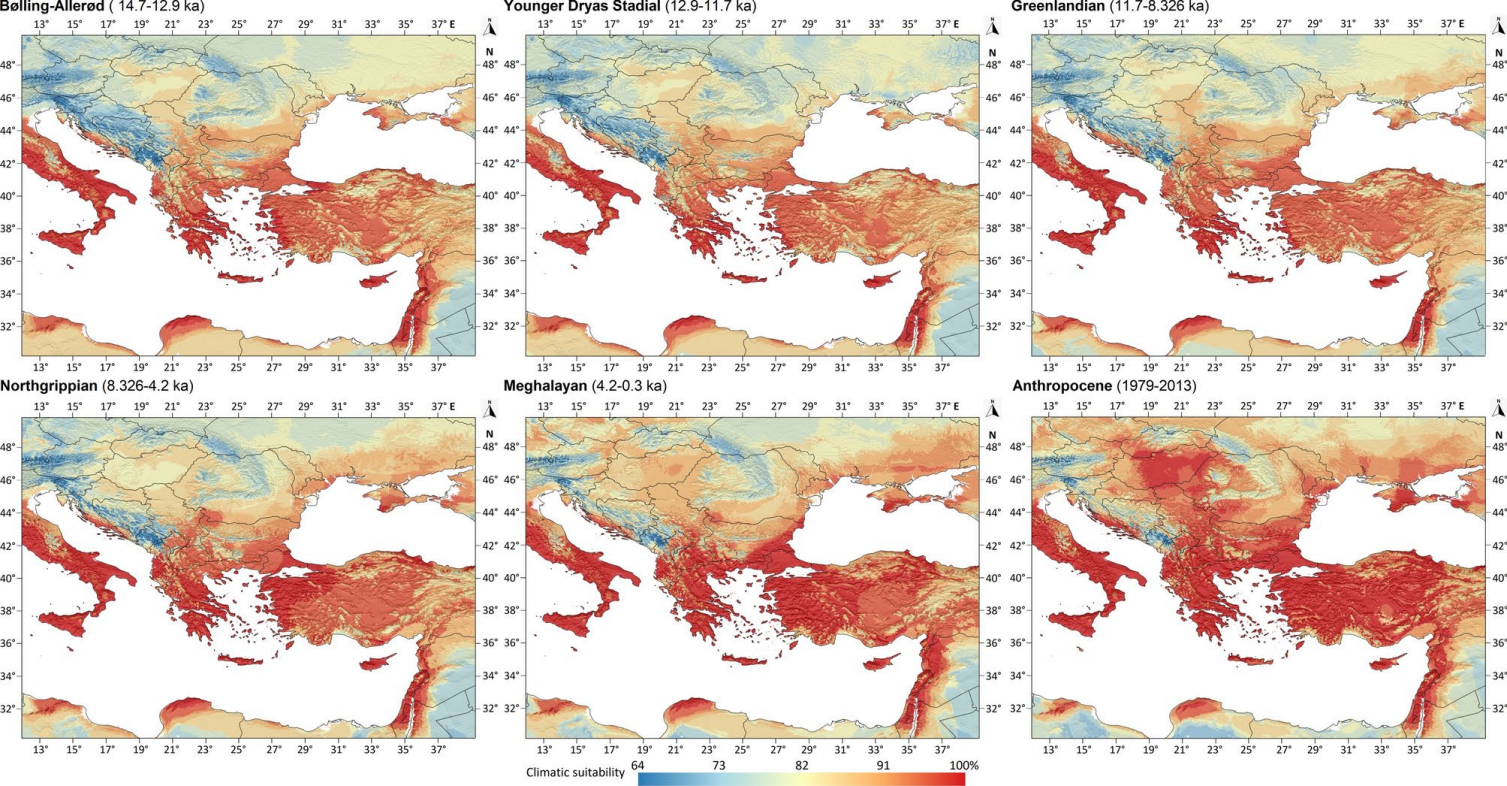
The climatic suitability patterns of *Ph. simci* in Southeast Europe and the Eastern Part of the Mediterranean Basin from the Bølling-Allerød to the Anthropocene. Ancient shorelines were not depicted on the maps.

Between the Bølling-Allerød (14.7–12.9 kya) and the Greenlandian stages (11.7–8.326 kya) (Fig. 5a–c), the potential climatic suitability values showed no notable range expansion compared to the above-presented Heinrich Stadial 1. The spread of *Ph. simici* could have started in the Northgrippian (8.326–4.2 kya) (Fig. 5d), and the rate of the territorial expansion of the suitable regions notably increased between the Meghalayan (4.2–0.3 kya) and the Anthropocene stages (Fig. 5e,f). In the studied region, currently the high climatic suitability regions of *Ph. simici* cover almost the entire Balkans, Asia Minor, the Levant, and show increasing habitability tendency also towards the Carpathian Basin.

**Fig. 6:**
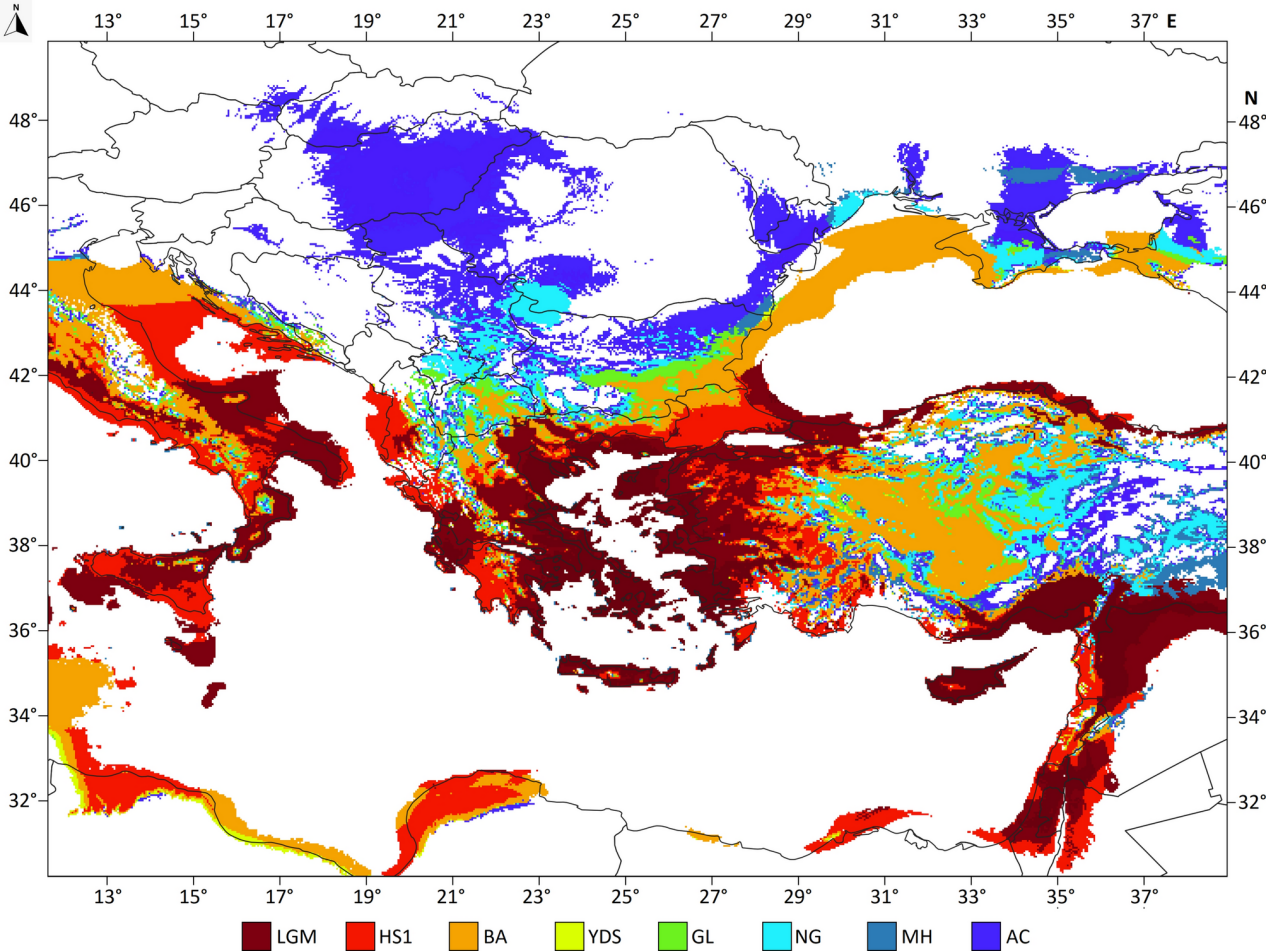
Changes of the 95% climatic suitable areas for *Ph. simici* between the Last Glacial maximum and the Anthropocene. The overlap of the colorized areas follows the Last Glacial Maximum (uppermost layer) to Anthropocene order. LGM: Last Glacial Maximum (lowermost layer), HS1: Heinrich Stadial 1 (17.0–14.7 kya), BA: Bølling-Allerød (14.7–12.9 kya), YDS: Younger Dryas Stadial (12.9–11.7 kya), GL: Greenlandian (11.7–8.326 kya), NG: Northgrippian (8.326–4.2 kya), MH: Meghalayan (4.2–0.3kya), AC: Anthropocene (1979–2013). The map was created by QGIS 3.34.11 with GRASS GIS v.7.6.1 (https://grass.osgeo.org) using country borders from Natural Earth (https://www.naturalearthdata.com).

The 95% climatic suitability areas show that around the Last Glacial maximum’s peak, the climatic suitability of *Ph. simici* were stable and mainly restricted to the per-Aegean region and the Levant. However, the potential spread of the species could have been slow until the end of the Younger Dryas Stadial. In the Holocene, the most rapid enlargement of the suitable climate habitats can be observed only in the last some hundred years (Fig. 6).

## Discussion

Despite excluding some *Adlerius* species such as *Ph. kabulensis*, *Ph. comatus*, or *Ph. fengi* due to a lack of available sequence data, this study provides the most comprehensive phylogenetic and divergence time analyses of the subgenus *Adlerius* based on the frequently used and readily available marker gene cytochrome oxidase subunit I (COI), which should support our understanding of diversification of this understudied subgenus. Additionally, we phylogeographically assessed the emergence of three geographically distinct lineages of *Phlebotomus simici*, a suspected vector for *L. infantum*, combined with climate suitability modeling to elucidate its post-glacial spread and potential current distribution.

Inferred divergence times were based on substitution rates of 1.0% and 2.5%/my, a minimum and maximum of a range that covers the rates typically inferred from/employed for mitochondrial protein coding genes in insects^28–30^. Phylogenetic relationships resembled the presently known geographic distribution of the analyzed species and provided insights into their divergence. Interestingly, the first major clade, which includes the easternmost species *Ph. simici* along with *Ph. brevis* and an unidentified *Adlerius* species from Türkiye and Armenia, diverged early (10 mya based on 2.5% and 25 mya based on 1.0% substitution rate) from the second major clade. This was also observed by Pavlou et al.^20^, who calculated a Cytb-based split of 8.79 mya between *Ph. simici* and all other analyzed *Adlerius* species.

The diversification of species comprising major clade II likely followed a western direction of dispersal, with the Chinese sand fly species *Ph. chinensis* and *Ph. sichuanensis* representing the geographically easternmost clade. The ancestor of *Ph. longiductus* and *Ph. turanicus* potentially lived on the southeastern coasts of the Parathetys Sea. However, it is currently difficult to find a climatically and geographically analogous area in relation to the late Miocene Central Paratethys. As it was shown, an ancestral species with similar climatic needs to present-day *Ph. simici* could have occupied the coasts and peninsulas of the Pannonian Lake. Another study reported similar findings for other Western Eurasian sand fly taxa of three other subgenera, namely *Larroussius*, *Paraphlebotomus* and *Phlebotomus*^22^. The joint analysis of weathering-produced sediments and paleoclimatic reconstructions indicates that the coastal regions of the Pannonian Lake had subtropical climatic conditions similar to the subtropical regions of the eastern United States or Southeast China^31^. Currently, large parts of the Japanese archipelago have similar climatic and geographical features, but, possibly due to the relative isolation of the Japanese archipelago from the mainland, it is home to only one sand fly species, *Sergentomyia squamirostris*^32^.

The geographical split between those two clades is very well visible between *Ph. chinensis* inhabiting central and eastern parts of China and *Ph. longiductus* being endemic in the north-eastern part of the country^13^. *Phlebotomus halepensis* and *Ph. arabicus* existed in the middle eastern side of the Paratethys Sea and split around 6.25 to 2.5 mya based on calculated divergence times from the clade comprising *Ph*. aff. *balcanicus* from Azerbaijan, *Ph. kyreniae* and *Ph. balcanicus* from Türkiye as well as *Ph. balcanicus* from the Balkans, altogether resembling the geographically westernmost clade of major clade II. The projected splits of these clades within major clade II might be connected to the formation of the Eurasian Mountain belt, and the climatic and geographical changes in the former Paratethys area which extended from the Northern foreland of the Alps to the Aral Sea in the Oligocene–Miocene epoch, around 33.9–5 mya. Interestingly, the Neogene climatic changes and the Paratethys provided special Eurasian geographical conditions that deeply impacted the phylogeography and climatic needs of current *Transphlebotomus* species^33^.

Interestingly, almost parallel splits can be observed between different lineages, namely *Ph. turanicus* from *Ph. longiductus* and undescribed *Adlerius* sp. from Moldova, *Ph. arabicus* from *Ph. halepensis*, splits within the clade comprising *Ph*. aff. *balcanicus*, *Ph. balcanicus* and *Ph. kyreniae* as well as *Ph. brevis* + *Adlerius* sp. from *Ph. simici*, which coincide with the Messinian Salinity Crisis 5.96–5.33 mya and the concurrent reflooding, which was also shown to be an important event for the diversification of *Transphlebotomus* species in the Aegean region^34^.

In major clade II, some species urge for taxonomic attention and clarification. Firstly, the yet undescribed *Adlerius* species from Moldova^11^ was observed to be the sister species of *Ph. longiductus* + *Ph. turanicus* and potentially split around 6.25 to 2.5 mya. Considering the western Asian and Middle eastern distribution of *Ph. turanicus*^35,36^ and the presence of *Ph. longiductus* in e.g. Kazachstan^37^ and as far west as Ukraine^38^, the species from Moldova likely followed a dispersal north of the Black Sea. Secondly, the clade including *Ph.* (aff.) *balcanicus*, *Ph. creticus* and *Ph. kyreniae* is of high taxonomic importance, as *Ph. balcanicus* is a suspected vector of *L. infantum* causing visceral leishmaniasis (VL) in Georgia^17^. In our analysis, we discriminated three distinct lineages that include *Ph. balcanicus* and *Ph*. aff. *balcanicus*. The species affinis (*Ph*. aff. *balcanicus*) found in Azerbaijan was shown to be the sister species of *Ph. kyreniae* and should thus be regarded as a distinct species. Also, *Ph. balcanicus* collected from Türkiye^4^ forms a distinct lineage as the sister species of *Ph. balcanicus* including only specimens from Balkan countries^5^ + *Ph. creticus*, which has so far been recorded only on the Greek Aegean island of Crete^9^. Similar in this respect to *Ph. simici*, *Ph. balcanicus* is a species with two genetically and geographically distinct lineages that cannot be discriminated morphologically. Based on geographic distribution, *Ph. balcanicus* in Georgia and Türkiye might represent the same lineage, however, no sequence data from Georgia is currently available to test this hypothesis.

Similarly to clade II, species of major clade I likely followed a westward dispersal. This is highlighted by the present-day distribution range of *Ph. simici*, *Ph. brevis* and undescribed *Adlerius* sp. from Middle Eastern countries to South-East Europe and supported by our Tortonian (11.608–7.246 mya) climatic suitability model that showed hypothetical climatic suitability for a *Ph. simici*-like ancient sand fly species as far as the southeastern part of central Europe, long coastal areas of the Pannonian Lake, the Balkan Peninsula except for the Dinarides and certain Aegean Lowland regions, Crete, the Bulgarian Lowland and ranges, large regions of Asia Minor, certain Levantine regions of the Middle East and the present-day Northern Cyrenaica region of Libya in North Africa.

Finally, our analysis provides strong support for the diversification of the three distinct *Ph. simici* lineages. The first and older split, 1.3 to 3.3 mya, between lineage I (Balkan) and the other two clades, lineage II (Israel) + lineage III (Türkiye), well correlates with the end-Neogene climatic changes including aridification and the increasing fluctuation of the climatic conditions in the Northern Hemisphere. Here, the climatic change between the mid-Pliocene warm (3.3 mya) and cold (3.205 mya) periods potentially supported the diversification since the glacial-interglacial alterations caused severe habitat contractions during the glacial phases and notable geographical extensions during the interglacial periods. Later, in the late Pleistocene, climatic changes caused similar habitat losses and gains for sand fly species in the Mediterranean^39^. Our modelled climatic suitability for *Ph. simici* in the study region changed considerably between these two study periods, however, climatic suitability was stable in coastal areas of south-western Türkiye. Particularly, in the warm period parts of northern Türkiye, mainland Greece and southern Balkan became climatically suitable and thus promoted the further western spread of *Ph. simici*. This coincides with our spatial diffusion analysis, which suggested the most recent common ancestor of *Ph. simici* in southwestern Türkiye and a consequent westward spread of lineage I. It should also be noted that the effect of mid-Pliocene warming could not be uniformly favorable for sand fly species in Europe, since models show that others, such as *Phlebotomus neglectus* or *Phlebotomus papatasi*-like ancient sand fly species, could suffer notable habitat losses due to the more humid climate during certain warm periods^40^. The second and younger split between lineage II and lineage III, 0.8 to 2.0 mya, and likely the diversification of different haplotypes in Türkiye as projected by our spatial diffusion analysis can be correlated to the start of the first glacial events in the early Pleistocene. This abrupt climatic change might coincide with the increasing extremes of climate, which resulted in, among others, the formation of mega-yardangs in central Europe and the appearance of such steppe-dwelling taxa like camelid and struthionid bird species in regions that previously had humid subtropical climate^41^.

Based on our phylogeographic analyses, we observed similar low levels of intra-clade divergence in lineage I (Balkan) and lineage II (Israel) with very recent population expansion, which is supported by the climatic models that showed high climatic suitability during the Last Glacial Maximum (LGM) only in southernmost part of the Balkans (e.g. now southern North Macedonia), coastal Greece including Aegean islands, and small regions of southern Israel. After the last Glacial period, a considerable change in climatic suitability was only noticed from the Northgrippian (8.326–4.2 kya) and after, supporting likely recent post-glacial expansion of *Ph. simici*. Despite having another distribution area, a pattern similarly projected for the post-glacial expansion of *Phlebotomus mascittii*^18^. Interestingly, expansion of the Turkish lineage (III) started well before the LGM between 200 and 700 kya, which is also very well supported by large areas of high climatic suitability during the Chibanian/MIS19 (787 kya) and last interglacial (130 kya) periods in Türkiye. Contrary to the phylogeographic data indicating little impact of LGM on this lineage, our climate models suggest limited climatic suitability in Türkiye during the LGM, with only parts of western Türkiye as well as small regions of coastal northern and southern parts of the country, however, several more microclimatic areas might have been suitable during LGM. This apparent contradiction can be resolved by noting that, although generally relatively small coastal areas remained suitable habitats for *Ph. simici* during the Last Glacial Maximum in the East Mediterranean Region, Figure 6 shows that areas of the eastern Aegean Archipelago and western Asia Minor that are now largely submerged under the sea could have also served as glacial refugia for the species during the Last Glacial Maximum.

Noteworthy, since the first and only record of *Ph. simici* in Austria in 2018^10^, this species was despite multiple trapping efforts never caught again at the same trapping site or any other locality surveyed in Austria for presence of sand flies. Likewise, the species has never been found in any of the bordering countries, albeit it is to note that except for sustained surveillance in Italy and Slovenia, only sporadic and localized surveys were performed there. Our phylogeographic analyses suggested a divergence of the Austrian sample from Balkan samples 60–150 kya. This does not necessarily mean that Austrian *Ph. simici* diverged from their Balkan conspecifics at that time but more likely indicates that we have not yet sampled all haplotypes present in the Balkan region or that the haplotype recorded in Austria is extinct there. However, the (rare) presence of *Ph simici* in Austria leaves open questions about its dispersal. While we modelled current potential but patchy climatic suitability as far north as small areas of eastern Austria (including the trapping location), southern Czech Republic, eastern Slovakia, and large parts of Hungary, climatic suitability was only shown after the late-Meghalayan for the recent Anthropocene reference period. Considering the absence of *Ph. simici* in Hungary^42^ and western Croatia^5^ as well as its potentially slow active dispersal ability^43^, anthropogenic dispersal should be taken into account. For example, Vaselek et al.^44^ investigated sand fly diversity along clusters of main transit and migration routes in Serbia and found significant changes of the sand fly fauna in Serbia during the end of the 20^th^ and beginning of the 21^st^ Century. However, the role of anthropogenic dispersal for sand flies remains unclear and calls for further elucidation.

## Conclusion

Our study provides valuable phylogenetic insights into the diversification and geographic appearance of the taxonomically complex and yet understudied subgenus *Adlerius*. We highlight that particularly suspected vector species forming intra-species lineages such as *Ph. balcanicus* and *Ph. simici* are of high medical and veterinary relevance and the emergence and involvement of various lineages in transmission should be carefully assessed.

The combination of phylogeographic analyses and climate modelling revealed a detailed understanding of diversification of three distinct *Ph. simici* lineages and their spread. Particularly, for the lineage comprising the Austrian specimen, the southern Balkan and coastal Greece formed an important glacial refugial area. The combination of the currently known distribution of *Ph. simici* and the modelled climatic suitability in Central Europe urges for further studies on the involvement of anthropogenic and natural factors in sand fly dispersal and spread. Our data clearly highlights the impact of changing climatic conditions to shifts of sand fly distribution and might help to better understand future dispersal connected to climate change, particularly in combination with, if feasible, deep population genetic analyses.

## Material and methods

### Sequence dataset, alignment and editing

For this study, available sequences obtained from GenBank as well as previously unpublished *Ph. simici* sequences were used (Supplementary Table 2). Amplification and sequencing of new specimens followed the protocol previously published by Studentsky et al.^45^. All sequences were aligned with ClustalX^46^, edited in GeneDoc 2.7^47^ and translated to amino acid sequences to assure intact reading frames and the absence of internal stop codons. The final alignment consisted of 168 individual *Adlerius* sequences with a length of 655 base pairs (bp), missing nucleotides were replaced with “N”.

### Phylogenetic relationships of *Ph. simici* and temporal patterns of diversification of the subgenus *Adlerius*

Phylogenetic relationships were inferred employing Maximum Likelihood (ML) and Bayesian (BI) approaches in IQ-TREE^48^ and MrBayes 3.2.6^49^ plugins of the PhyloSuite 1.2.2 package^50^. The best fitting model of evolution to be used in phylogenetic tree inference was selected based on the Bayesian information criterion (BIC) in Modelfinder^51^, as implemented in PhyloSuite. A standard ML tree search was conducted in IQ-TREE and node support was assessed with 1000 standard bootstrap replicates. For Bayesian phylogenetic inference with MrBayes, posterior probabilities were obtained from Metropolis-coupled Markov chain Monte Carlo (MCMCMC) simulations (2 independent runs, 6 chains, 10 million generation, sampling frequency 1000, 25% burn-in). Chain stationarity and parameter convergence (effective sample sizes (ESS) > 200, indicating that the parameter log file accurately reflects the posterior distribution^52^; were checked in Tracer 1.7^53^ and via monitoring the average standard deviation of split frequencies (< 0.01). Post burn-in trees were summarized in a 50% majority rule consensus tree. Visualization of ML and BI trees was done in FigTree v.1.4.2 (available at http://tree.bio.ed.ac.uk/software/figtree).

In addition, we inferred a chronogram in BEAST2.7.3^54^. Prior to this analysis, the originally 168 sequences were collapsed into 109 haplotypes using FaBox 1.61^55^. We employed the best fitting model of evolution as suggested by Modelfinder, an optimized relaxed-clock model, a substitution rate of 0.01 and 0.025 substitutions per site per million years (my)^28,56^, which covers the range of substitution rates typically inferred from/employed for mitochondrial protein coding genes in insects^29,30,57,58^, and a birth–death tree prior. We ran three independent MCMC runs for 150 million generation each and sampled parameters every 1000 generation. LogCombiner (part of the BEAST2 package) was then used to combine the three runs after discarding the first 10% of generations as bun-in. Chain stationarity and parameter convergence were assessed in Tracer. Pooled post-burn-in ESS was > 200 for all parameters. A maximum clade credibility tree was computed from the post-burn-in trees using TreeAnnotator (part of the BEAST2 package) and visualized in FigTree.

### Genetic diversity of *Ph. simici* through space and time

DnaSP v.5^59^ was used to calculate unique haplotypes, haplotype (Hd), and nucleotide (π) diversity. Uncorrected pairwise distances between all species and all *Ph. simici* haplotypes were calculated in MEGAX^60^. Phylogenetic relationships among *Ph. simici* haplotypes were visualized as a statistical parsimony network^61^ inferred in PopART^62^. To test for signatures of recent population expansion in major clades of *Ph. simici*, we calculated mismatch distributions (1,000 bootstrap replicates) and the neutrality test statistics Tajima’s D (^98^; 10,000 simulated samples) and Fu’s Fs (^99^; 10,000 simulated samples) in Arlequin 3.5.2.2^63^. The fit between the observed mismatch distribution and expectations based on growth parameter estimates was evaluated by the sum of squared differences (SSD) and the raggedness index (rg). In addition, we inferred past population size changes by means of Bayesian skyline plots (BSPs) in BEAST 2.7.3. We employed the best-fitting models of molecular evolution selected by the BIC in Modelfinder, and a strict molecular clock with again a minimum and maximum substitution rate of 0.01 and 0.025 substitutions per site per my, and the Bayesian Skyline tree prior. Four independent MCMC runs of three to twelve million generations (depending on dataset size; adjusted after preliminary runs), each were done, sampling every 1000th step and a burn-in of the first 10% of sampled generations. LogCombiner was used to combine the individual log and tree files. Assessment of run convergence (ESS > 200 for all parameters), and visualization of past population size changes were done in Tracer.

Finally, the species’ spatial history was assessed employing a cauchy relaxed random walk (RRW) diffusion model implemented in BEAST 1.10.4^64^. We used a normally distributed diffusion rate, Bayesian Skyline tree prior, the best-fitting modes of molecular evolution selected by the BIC in Modelfinder, a strict molecular clock with again minimum and maximum substitution rates of 0.01 and 0.025 substitutions per site per my, and a Bayesian Skyline coalescent tree prior. The jitter option was set to 0.01 to create noise in identical coordinates. Runs were run for 300 million generations, sampled every 1000th step and with a burn-in of the first 10% of the sampled generations. Assessment of run performance (ESS > 200 for all parameters) was done in Tracer. A maximum clade credibility obtained in TreeAnnotator (part of the BEAST1 package) was used as input for EvoLaps2 v. 2.42^65^ (https://www.evolaps.org) to visualize the spatiotemporal pattern.

### Geographical data and mapping

A literature search for records of *Ph. simici* was performed (Google Scholar and PubMed), and coordinates or locations were extracted^4,5,9,10,16,44,66–82^. In addition, a dataset acquired in the frame of the VectorNet project was exploited for presence/absence data^83^. Coordinates of trapping sites were georeferenced into a distribution map using Quantum GIS 3.4.11^84^ (Supplementary Fig. 3).

### Climatic and topographic data sources

The climatic classification of the *Ph. simici* sites was determined using the Köppen–Geiger system. For this purpose, the present-day Köppen–Geiger map produced by Beck et al.^85^ was used. A total of one climatic reference period and twelve paleoclimatic data sources were used, covering a period spanning from the early Late Miocene to the Anthropocene. By epochs, one data source provided the Tortonian paleoclimatic data, two models represent the mid-Pliocene epoch, one model covers the mid-Pleistocene (the MIS19 interstadial), five models belong to the “Late” Pleistocene, three models represent the Holocene, and one the Anthropocene, which forms the reference period in this study (Supplementary Table 3). Topographic data were sourced from the ETOPO Global Relief Model^86^.

The Tortonian climatic model was produced by the paleoclimatic reconstruction of Bruch et al.^87^. It contains paleoclimatic reconstructions by fossil sites based on the coexistence analysis of fossil plant assemblages. This approach is used for the quantitative reconstructions of the Tertiary terrestrial paleoclimates. It is based on the idea that tertiary plant taxa require similar climates to those of their closest extant relatives^88^. It is a frequently used method to produce paleoclimatic reconstructions for the Cenozoic period, including even the Paleogene period^89^. To create the Tortonian paleoclimatic model for Europe and the neighboring regions (see details in Supplementary Table 4), the following steps were conducted:

1. The Tortonian plant fossil sites were georeferenced with the corresponding paleoclimatic data in the attribute table using QGIS version 3.8.3 with GRASS GIS version 7.6.1.
2. After that the differences between the Tortonian palaoclimatic^87^ and the reference period’s^90^ climatic values were calculated.
3. In the next step, the calculated difference values were georeferenced byclimatic factors, and the point-like difference values were IDW-interpolated.
4. In the final step, the original reference period’s values were modified by the IDW-interpolated values.

### IDW interpolation of paleoclimatic values

As mentioned before, to produce paleoclimatic maps for the Tortonian, an Inverse Distance Weighting (IDW) method was used in QGIS^91^, which is a spatial interpolation method used to estimate values at unsampled locations based on values from nearby sampled points. The core idea of IDW is that points closer to the estimation location have more influence on the predicted value than points further away. This method assumes that the influence of the interpolated variable decreases with distance, hence the term “inverse distance” (Supplementary Fig. 4). IDW calculates the value at an unsampled location as a weighted average of the values of nearby points. The weights are inversely proportional to the distance between the known points and the point being estimated, giving more influence on closer points. The influence of the distance is controlled by a power parameter *p*. A higher *p* value increases the influence of nearer points and reduces the influence of farther points more sharply. The method typically uses a set number of nearest neighbors or a fixed radius to include surrounding points in the interpolation calculation.

The value *V*(*x*) at an unsampled location x is calculated using the following equation:

1 $$V\left( x \right) = \frac{{\mathop \sum \nolimits_{i = 1}^{n} \frac{{V_{i} }}{{d_{i}^{p} }}}}{{\mathop \sum \nolimits_{i = 1}^{n} \frac{1}{{d_{i}^{p} }}}}$$

where *V*(*x*) is the interpolated value at location *x*; *V*_*i*_ is the known value at the *i*-th sampled point; *d*_*i*_ is the distance between the unsampled location* x* and the *i*-th sampled point; *p* is the power parameter, typically a positive value that determines the weight of the distance (common values are between 1 and 3); *n* is the number of sampled points used for interpolation.

Because the original paleoclimatic reconstruction of Bruch et al.^87^ contains only four factors (the mean annual temperature, the mean temperature of the coldest months, the mean temperature of the warmest months, and the annual precipitation (sum), two additional factors were created based on the existing ones to enhance the precision of climatic suitability models:

2 $$MATR = Tm_{07} - Tm_{07}$$

where *MATR* is the mean annual temperature range, *Tm*_*07*_ and *Tm*_*01*_ are the mean July and January temperature. The unit is °C.

This derived factor characterizes the fluctuations of the thermal conditions during the year, which can be an important factor for sand fly seasonality and occurrence^92^.

In addition, the Thornthwaite Agrometeorological Index^93^ was introduced to characterize the general humidity conditions which is also an important factor for sand fly activity and occurrence^94^. The applied equation was as follows based on the modified equation of^95^:

3 $$TAI = 1.65 \times \frac{{\frac{bio12}{{12}}}}{bio1 + 12.2}^{\frac{10}{9}}$$

where *TAI* is the Thornthwaite Agrometeorological Index, *bio1* is the mean annual temperature, *bio12* is the annual precipitation sum. The unit is mm °C^−1^.

### Determination of lower and upper extrema

Climate values at *Ph. simici* occurrence points were sampled across 16 bioclimatic layers (2 monthly and 14 bioclimatic maps) using the QGIS sampling tool. To refine the data, the highest and lowest 1% of values for each bioclimatic variable were excluded before determining the upper and lower extremes. This process ensured sampling based on the known occurrences of *Ph. simici* and improved data quality.

Formally, let *X* be the set of climate values sampled at occurrence points across *n* climatic variables {*X*_*1*_*, X*_*2*_*,…,X*_*n*_}. For each variable *X*_*i*_, let *X*_*i*_^(sorted)^ represent the ordered values of *X*_*i*_.

To determine the purified data for each variable *X*_*i*_:

4 $$X_{i}^{cleaned} = X_{i}^{{\left( {sorted} \right)}} \left[ k \right],\;X_{i}^{{\left( {sorted} \right)}} \left[ {k + 1} \right], \ldots ,\;X_{i}^{{\left( {sorted} \right)}} \left[ {m - k} \right]$$

where k = ⌈0.01 × m⌉ and *m* is the total number of samples in *X*_*i*_.

### Model identification: modelling of climatic suitability patterns

Climate Envelope Method (CEM) forms the basic concept of the creation of climatic suitability methods. Climate envelope modelling is an approach used to predict the potential distribution of a species or phenomenon based on the climatic conditions that are currently associated with its presence^96^. This method assumes that the current distribution of a species is largely determined by a set of climatic variables (e.g. temperature, precipitation) that fall within specific upper and lower thresholds suitable for the species. By defining these climatic “envelopes,” or ranges, climate envelope models can be used to predict where the species could potentially exist under different climate scenarios.

The mathematical basis of CEM is the Boolean algebra^97^. In Boolean climate envelope modelling, the presence or absence of a species in a given area is determined by whether the climatic conditions fall within the specified limits for each variable. The presence-absence maps for each climatic factor are combined using Boolean algebra to generate an overall suitability map. The general Boolean equation for the climate envelope model can be expressed as:

5 $$S\left( x \right) = \bigwedge_{i = 1}^{n} \left( {L_{i} \le V_{i} \le U_{i} } \right)$$

where *S*_*x*_ is the overall suitability (1 for presence, 0 for absence) at location, *n* is the number of climatic variables which is 6 in the case of the Tortonian and 14 in the case of the Pliocene, Pleistocene and Holocene models (see Supplementary Table 3); *V*_*i*_(*x*) is the value of the *i*-th climatic variable at location *x*; *L*_*i*_ and *U*_*i*_ are the lower and upper limits for the *i*-th climatic variable; ⋀ represents the logical AND operation.

Each variable’s presence-absence condition is defined as 1 (suitable) if *Li* ≤ *Vi*(*x*) ≤ *Ui*, otherwise 0 (unsuitable). The overall suitability map is obtained by the intersection (logical operator AND, ⋀) of all these individual presence-absence maps. There were three kinds of climatic models: temperature like (T), precipitation-like (P), and humidity-like (H) ones.

Two model environments were built: one for the Tortonian and another one for the other climatic suitability models. The model equation for the Tortonian is as follows:

6 $$S\left( x \right) = \left( { \bigwedge_{i = 1}^{4} \left( {L_{Ti} \le T_{i} \left( x \right) \le U_{Ti} } \right)} \right) \bigwedge \left( {L_{P1} \le P_{1} \left( x \right) \le U_{P1} } \right) \bigwedge \left( {L_{H1} \le H_{1} \left( x \right) \le U_{H1} } \right)$$

where *S*(*x*) is the overall climatic suitability at location *x*; *T*_*i*_(*x*) is the value of the *i*-th temperature factor at location *x*; *P*_*1*_(*x*) is the value of the precipitation-like factor at location *x*; *H*_*1*_(*x*) is the value of the humidity-like factor at location *x*; *L*_*Ti*_ and *U*_*Ti*_ are the lower and upper limits for the *i*-th temperature-like factor; *L*_*P1*_ and *U*_*P1*_ are the lower and upper limits for the precipitation-like factor; *L*_*H1*_ and *U*_*H1*_ are the lower and upper limits for the humidity-like factor. ⋀ represents the logical AND operation.

This equation ensures that the suitability at a location *x* is determined only if all four temperature-like factors, the precipitation-like factor, and the humidity-like factor fall within their respective suitable ranges. The model equation for the Pliocene, Quaternary, and Holocene periods is as follows:

7 $$S\left( x \right) = \left( { \bigwedge_{i = 1}^{6} \left( {L_{Ti} \le T_{i} \left( x \right) \le U_{Ti} } \right)} \right) \bigwedge \left( { \bigwedge_{j = 1}^{8} \left( {L_{Pj} \le P_{i} \left( x \right) \le U_{Pj} } \right)} \right)$$

where *S*(*x*) is the overall climatic suitability at location *x*; *T*_*i*_(*x*) is the value of the *i*-th temperature factor at location *x*; *P*_*j*_(*x*) is the value of the *j*-th precipitation-like factor at location *x*; *L*_*Ti*_ and *U*_*Ti*_ are the lower and upper limits for the *i*-th temperature factor; *L*_*Pj*_ and *U*_*Pj*_ are the lower and upper limits for the *j*-th precipitation-like factor.

This above-presented equation combines the suitability conditions for all temperature and precipitation-like factors to produce a final climatic suitability map. The species or phenomenon is considered present at a location *x* only if all the climatic variables fall within their specified suitable ranges simultaneously. Supplementary Table 5 shows the used climatic factors according to the two model environments.

## Supplementary Information

Supplementary Figure 1.

Supplementary Figure 2.

Supplementary Figure 3.

Supplementary Figure 4.

Supplementary Table 1.

Supplementary Table 2.

Supplementary Table 3.

Supplementary Table 4.

Supplementary Table 5.

## Data Availability

All data are included in the article and supplementary material.
