# Supplementary material for: Combining phylogeography and climate models to track the diversification and spread of Phlebotomus simici
Source: Sci Rep. 2025 Mar 25;15:10188. doi: 10.1038/s41598-025-94601-1 (PMC11933271; doi:10.1038/s41598-025-94601-1)
Supplement: Supplementary file 1 — Supplementary Figure 1. [file 41598_2025_94601_MOESM1_ESM.docx]

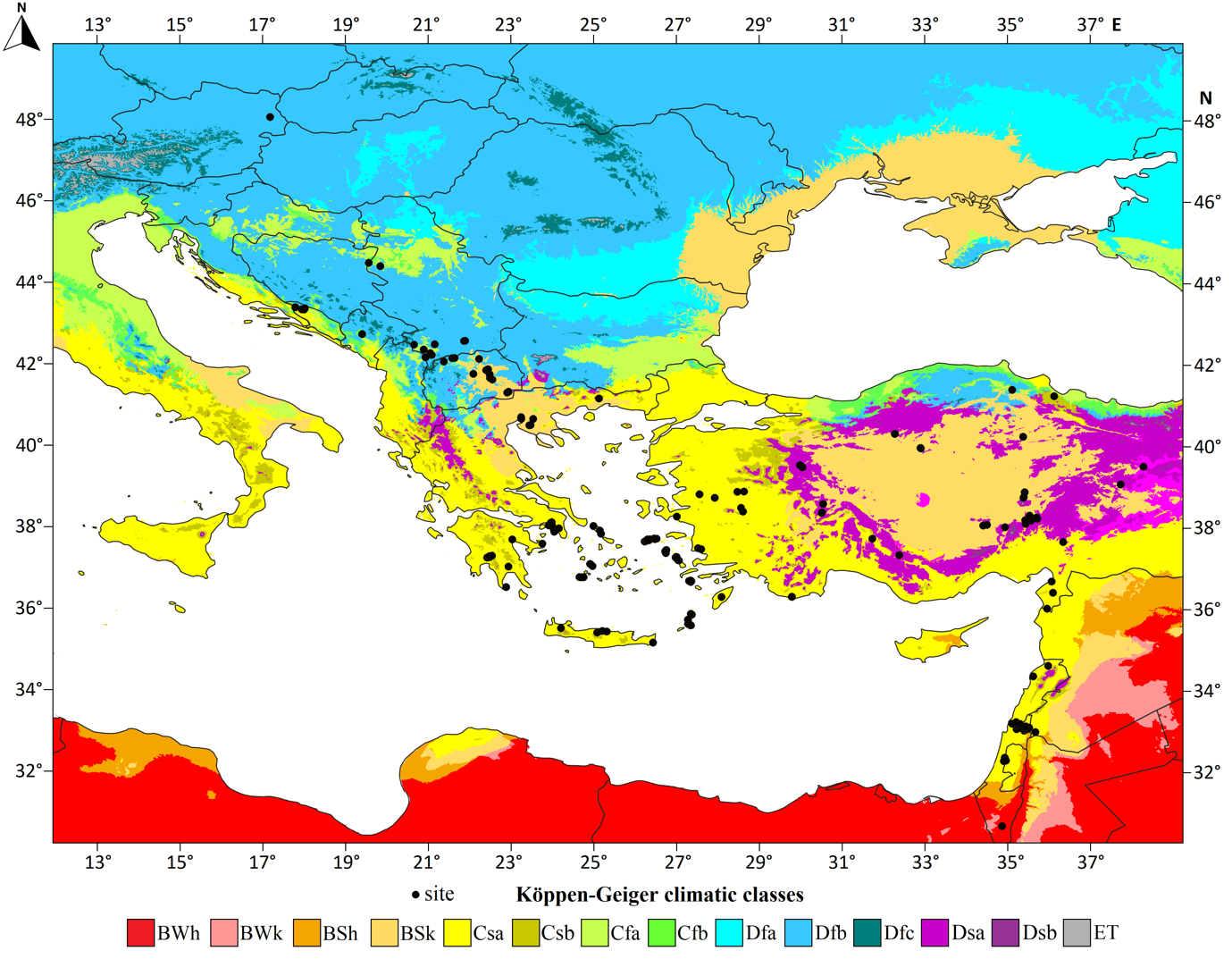


**Supplementary Figure 1**. The studied *Ph. simici* sites projected to the reference period’s Köppen-Geiger model. BWh: hot desert climates, BWk: cold desert climates, BSh: hot semi-arid climates, BSk: cold semi-arid climate, Csa: hot-summer Mediterranean climate, Csb: warm-summer Mediterranean climate, Cfa: humid subtropical climate, Cfb: temperate oceanic climate, Dfa: hot-summer humid continental climate, Dfb: warm-summer humid continental climate, Dfc: subarctic climate, Dsa: Mediterranean-influenced hot-summer humid continental climate; Dsb: Mediterranean-influenced warm-summer humid continental climate; ET: Tundra climate.
