# Supplementary material for: Combining phylogeography and climate models to track the diversification and spread of Phlebotomus simici
Source: Sci Rep. 2025 Mar 25;15:10188. doi: 10.1038/s41598-025-94601-1 (PMC11933271; doi:10.1038/s41598-025-94601-1)
Supplement: Supplementary file 2 — Supplementary Figure 2. [file 41598_2025_94601_MOESM2_ESM.docx]

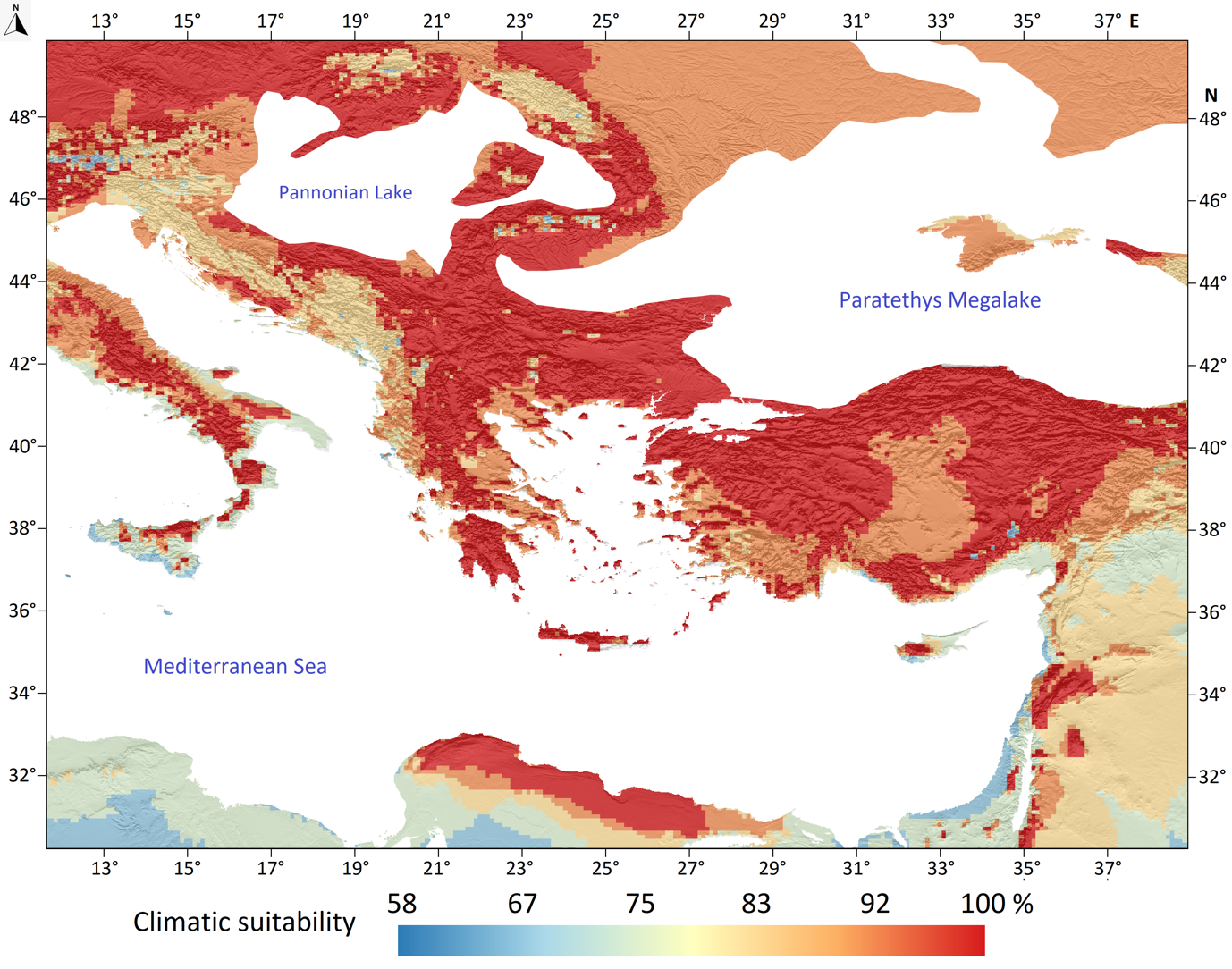


**Supplementary Figure 2**. The Tortonian (11.608–7.246 ma) climatic suitability patterns of a *Ph. simici*-ancestor species with similar climatic constraints as the present-day *Ph. simici* in Southeast Europe and the eastern part of the Mediterranean Basin.
