# Supplementary material for: Combining phylogeography and climate models to track the diversification and spread of Phlebotomus simici
Source: Sci Rep. 2025 Mar 25;15:10188. doi: 10.1038/s41598-025-94601-1 (PMC11933271; doi:10.1038/s41598-025-94601-1)
Supplement: Supplementary file 3 — Supplementary Figure 3. [file 41598_2025_94601_MOESM3_ESM.docx]

**Supplementary Figure 3.** Currently known *Ph. simici* distribution extracted from the literature. Elevation data used from ETOPO Global Relief Model (https://www.ncei.noaa. gov/products/etopo-global-relief-model).
