# Supplementary material for: Combining phylogeography and climate models to track the diversification and spread of Phlebotomus simici
Source: Sci Rep. 2025 Mar 25;15:10188. doi: 10.1038/s41598-025-94601-1 (PMC11933271; doi:10.1038/s41598-025-94601-1)
Supplement: Supplementary file 4 — Supplementary Figure 4. [file 41598_2025_94601_MOESM4_ESM.docx]

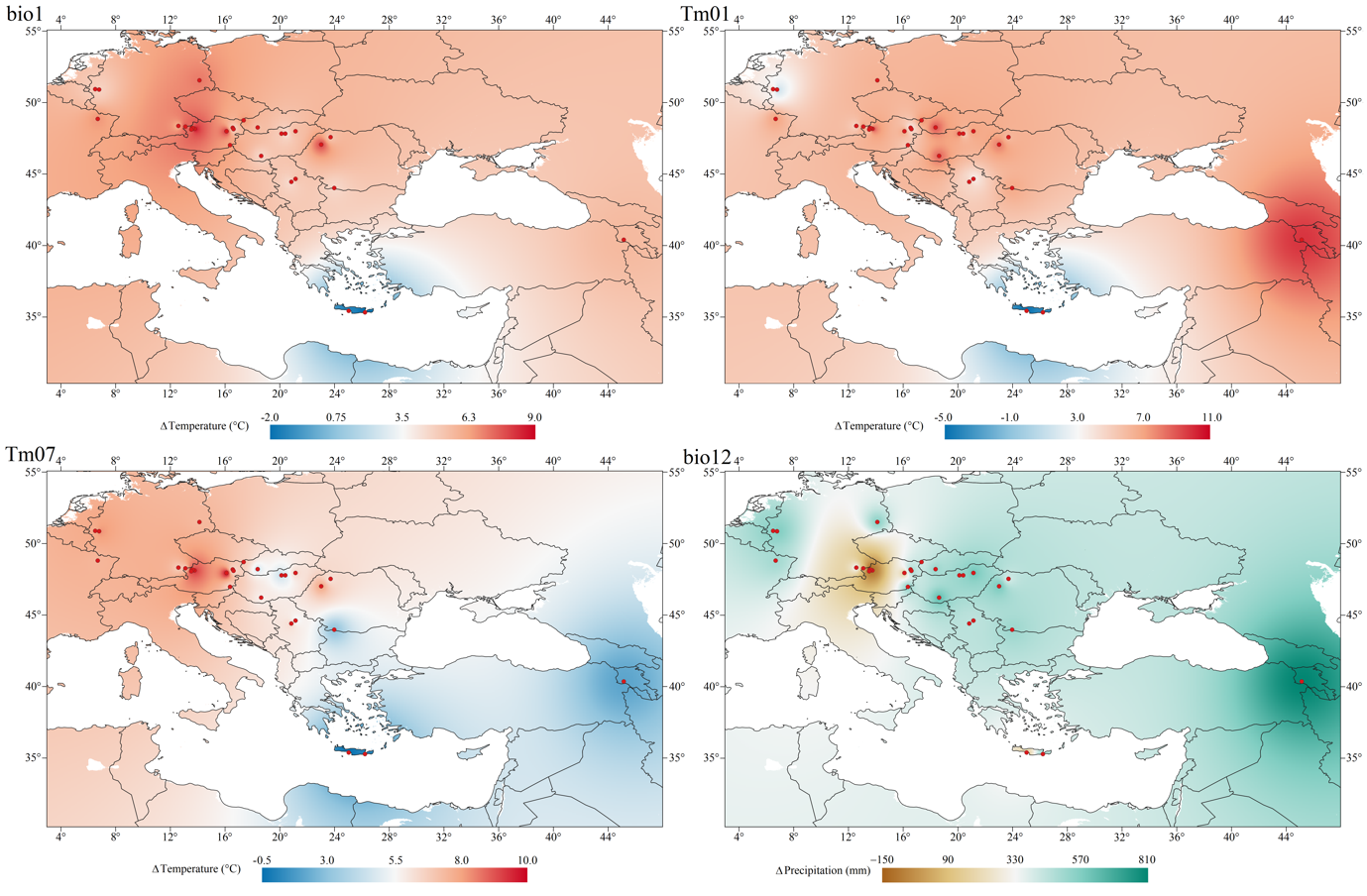


**Supplementary Figure 4**. The IDW interpolated difference values by climatic values. bio1: annual mean temperature, Tm01: mean temperature of the coldest month (January), Tm07: mean temperature of the warmest month (July), bio12: annual precipitation. The red points mark the sites of the fossil assemblages which formed the basis of the coexistence method-based reconstruction of the Tortonian paleoclimates in the study of Bruch et al.^89^.
