# Supplementary material for: Combining phylogeography and climate models to track the diversification and spread of Phlebotomus simici
Source: Sci Rep. 2025 Mar 25;15:10188. doi: 10.1038/s41598-025-94601-1 (PMC11933271; doi:10.1038/s41598-025-94601-1)
Supplement: Supplementary file 7 — Supplementary Table 3. [file 41598_2025_94601_MOESM7_ESM.docx]

**Supplementary Table 3**. Main data of the used climatic, paleoclimatic models.

| **No** | **Period** | **Epoch** | **Stage** | **Age** | **Resolution** | **Type** | **Reference** |
| --- | --- | --- | --- | --- | --- | --- | --- |
| 1 | Miocene | Tortonian | na | 11.608-7.246 Ma | point-like | site-based data | Bruch et al. (2007) |
| 2 | Pliocene | Piacenzian | M2 mid-Pliocene cold period | 3.3 Ma | 2.5 arc-min | mapped model | Dolan et al. (2015) |
| 3 | Pliocene | Piacenzian | mid-Pliocene warm period | 3.205 Ma | 2.5 arc-min | mapped model | Hill (2015) |
| 4 | Quaternary | Pleistocene | Chibanian, MIS19 | 787 ka | 2.5 arc-min | mapped model | Brown et al. (2018) |
| 5 | Quaternary | Pleistocene | “Late”, Last Interglacial | 130 ka | 2.5 arc-min | mapped model | Otto-Bliesner et al. (2006) |
| 6 | Quaternary | Pleistocene | “Late”, Last Glacial Maximum | 21 ka | 2.5 arc-min | mapped model | Karger et al. (2021) |
| 7 | Quaternary | Pleistocene | “Late”, Heinrich Stadial 1 | 17.0-14.7 ka | 2.5 arc-min | mapped model | Fordham et al. (2017) |
| 8 | Quaternary | Pleistocene | “Late”, Bølling-Allerød | 14.7-12.9 ka | 2.5 arc-min | mapped model | Fordham et al. (2017) |
| 9 | Quaternary | Pleistocene | “Late”, Younger Dryas Stadial | 12.9-11.7 ka | 2.5 arc-min | mapped model | Fordham et al. (2017) |
| 10 | Quaternary | Holocene | early-Holocene, Greenlandian | 11.7-8.326 ka | 2.5 arc-min | mapped model | Fordham et al. (2017) |
| 11 | Quaternary | Holocene | mid-Holocene, Northgrippian | 8.326-4.2 ka | 2.5 arc-min | mapped model | Fordham et al. (2017) |
| 12 | Quaternary | Holocene | late-Holocene, Meghalayan | 4.2-0.3 ka | 2.5 arc-min | mapped model | Fordham et al. (2017) |
| 13 | Quaternary | Anthropocene,  reference period | na | 1979-2013 | 2.5 arc-min | mapped model | Karger et al. (2021) |
