# Supplementary material for: Combining phylogeography and climate models to track the diversification and spread of Phlebotomus simici
Source: Sci Rep. 2025 Mar 25;15:10188. doi: 10.1038/s41598-025-94601-1 (PMC11933271; doi:10.1038/s41598-025-94601-1)
Supplement: Supplementary file 8 — Supplementary Table 4. [file 41598_2025_94601_MOESM8_ESM.docx]

**Supplementary Table 4**. The used primary Tortonian and present-day climatic data with the difference values. Site: fossil plant site. Lat: Latitude, Lon: Longitude; t prefix: Tortonian data, p prefix: present-day data; D: difference between the Tortonian and present-day climatic values. bio1: annual mean temperature, Tm01: mean temperature of the coldest month (January), Tm07: mean temperature of the warmest month (July), bio12: annual precipitation.

| **Site** | **Country** | **Lat** | **Lon** | **tbio1** | **tTm01** | **tTm07** | **tbio12** | **pbio1** | **pTm01** | **pTm07** | **pbio12** | **Dbio1** | **DTm01** | **DTm07** | **Dbio12** |
| --- | --- | --- | --- | --- | --- | --- | --- | --- | --- | --- | --- | --- | --- | --- | --- |
| Ampflwang | Austria | 13.55 | 48.1 | 17.75 | 8.9 | 27.05 | 1146.5 | 7.65 | -3.10 | 16.8 | 1224 | 10.10 | 12.00 | 10.25 | -77.50 |
| Aubenham | Germany | 12.4 | 48.3 | 14.3 | 2.1 | 24.05 | 1234 | 8.44 | -2.50 | 17.3 | 889 | 5.86 | 4.60 | 6.75 | 345.00 |
| Bükkábrány | Hungary | 20.75 | 47.92 | 15.5 | 4.35 | 26.9 | 1126 | 10.05 | -2.70 | 20.5 | 529 | 5.45 | 7.05 | 6.40 | 597.00 |
| Bulgaria | Bulgaria | 23.51 | 43.87 | 16.4 | 5.8 | 26 | 1065.5 | 11.81 | -0.90 | 22.9 | 555 | 4.59 | 6.70 | 3.10 | 510.50 |
| Delureni | Romania | 22.58 | 46.97 | 16.85 | 5.4 | 27.2 | 1239 | 9.14 | -2.50 | 19.4 | 660 | 7.71 | 7.90 | 7.80 | 579.00 |
| Dubona | Serbia | 20.45 | 44.31 | 14.9 | 4.25 | 26.6 | 1179.5 | 10.53 | -0.60 | 20.2 | 730 | 4.37 | 4.85 | 6.40 | 449.50 |
| Durinci | Serbia | 20.75 | 44.52 | 16.05 | 3.3 | 26.05 | 1120 | 10.77 | -0.10 | 20.7 | 675 | 5.28 | 3.40 | 5.35 | 445.00 |
| Frechen | Germany | 6.75 | 50.89 | 14.75 | 2.55 | 26.25 | 1279 | 10.04 | 1.60 | 17.9 | 709 | 4.71 | 0.95 | 8.35 | 570.00 |
| Grossenreith | Austria | 13.36 | 48.16 | 14.7 | 2.35 | 26.35 | 919 | 8.02 | -2.60 | 17.3 | 1096 | 6.68 | 4.95 | 9.05 | -177.00 |
| Hambach | Germany | 6.47 | 50.92 | 15.1 | 6.3 | 25.75 | 1240.5 | 10.12 | 2.10 | 18.2 | 707 | 4.98 | 4.20 | 7.55 | 533.50 |
| Hidas | Hungary | 18.3 | 46.16 | 15 | 6.6 | 26.2 | 1242.5 | 10.36 | -1.80 | 20.4 | 628 | 4.64 | 8.40 | 5.80 | 614.50 |
| Hoktemberyan | Armenia | 44.15 | 40.18 | 17 | 6.35 | 26.75 | 1104.5 | 11.42 | -3.40 | 24.7 | 294 | 5.58 | 9.75 | 2.05 | 810.50 |
| Klettwitz | Germany | 13.9 | 51.55 | 16 | 5.45 | 25.7 | 1167 | 9.05 | -0.90 | 18.3 | 629 | 6.95 | 6.35 | 7.40 | 538.00 |
| Laaerberg | Austria | 16.27 | 48.17 | 14.5 | 2.3 | 26 | 1042 | 9.71 | -1.00 | 19.5 | 579 | 4.79 | 3.30 | 6.50 | 463.00 |
| Leonberg | Germany | 12.9 | 48.25 | 16 | 5.25 | 23.95 | 1105 | 8.40 | -2.10 | 17.8 | 964 | 7.60 | 7.35 | 6.15 | 141.00 |
| Lohnsburg | Austria | 13.42 | 48.15 | 14.55 | 2.35 | 26.05 | 934 | 8.05 | -2.60 | 17.5 | 1083 | 6.50 | 4.95 | 8.55 | -149.00 |
| Makrilia | Greece | 25.7 | 35 | 16.8 | 8.65 | 26.55 | 988.5 | 18.85 | 12.70 | 25.6 | 507 | -2.05 | -4.05 | 0.95 | 481.50 |
| Moravian_Basin | Czech_Republic | 17.05 | 48.7 | 16.05 | 5.7 | 26.05 | 1097 | 9.61 | -1.70 | 19.3 | 685 | 6.44 | 7.40 | 6.75 | 412.00 |
| Neuhaus | Austria | 16.08 | 46.93 | 15.7 | 3.2 | 25.85 | 1293 | 9.42 | -1.90 | 19.3 | 767 | 6.28 | 5.10 | 6.55 | 526.00 |
| Neusiedl | Austria | 15.83 | 47.92 | 14.9 | 2.65 | 25.55 | 1126 | 6.69 | -4.40 | 15 | 984 | 8.21 | 7.05 | 10.55 | 142.00 |
| Nitra | Slovakia | 18.05 | 48.19 | 15.45 | 7.05 | 24.8 | 1055.5 | 10.05 | -1.80 | 19.9 | 542 | 5.40 | 8.85 | 4.90 | 513.50 |
| Oas_Basin | Romania | 23.25 | 47.5 | 14.8 | 3.55 | 26.05 | 1111.5 | 9.54 | -2.60 | 19.8 | 707 | 5.26 | 6.15 | 6.25 | 404.50 |
| Rózsaszentmárton | Hungary | 19.75 | 47.75 | 15.6 | 6 | 24.8 | 1024 | 10.37 | -2.10 | 20.4 | 520 | 5.23 | 8.10 | 4.40 | 504.00 |
| Schneegattern | Austria | 13.32 | 48.04 | 14.75 | 2.3 | 26.75 | 1167 | 7.55 | -2.70 | 17.1 | 1220 | 7.20 | 5.00 | 9.65 | -53.00 |
| Triopetra | Greece | 24.54 | 35.11 | 17 | 7.2 | 26.25 | 747 | 18.48 | 12.00 | 25.7 | 566 | -1.48 | -4.80 | 0.55 | 181.00 |
| Visonta | Hungary | 20.02 | 47.75 | 14.55 | 2.55 | 25.6 | 1051.5 | 10.45 | -2.10 | 20.6 | 504 | 4.10 | 4.65 | 5.00 | 547.50 |
| Voesendorf | Austria | 16.33 | 48.07 | 14.85 | 3.95 | 26.1 | 1066 | 10.10 | -0.80 | 19.9 | 563 | 4.75 | 4.75 | 6.20 | 503.00 |
| Wien | Austria | 6.65 | 48.8 | 16.1 | 7.55 | 25.6 | 1293 | 9.64 | 0.70 | 18.1 | 798 | 6.46 | 6.85 | 7.50 | 495.00 |
