# Supplementary material for: Combining phylogeography and climate models to track the diversification and spread of Phlebotomus simici
Source: Sci Rep. 2025 Mar 25;15:10188. doi: 10.1038/s41598-025-94601-1 (PMC11933271; doi:10.1038/s41598-025-94601-1)
Supplement: Supplementary file 9 — Supplementary Table 5. [file 41598_2025_94601_MOESM9_ESM.docx]

**Supplementary Table 5**. The used climatic factors by model environments in climatic suitability modelling processes. T: temperature-like, P: precipitation-like, H: humidity-like climatic factors.

| **Climatic factor** | **Abbreviation** | **Unit** | **Factor type** | **Model use** | **Lower limit (L)** | **Upper limit (U)** |
| --- | --- | --- | --- | --- | --- | --- |
| January mean temperature | Tm_01_ | °C | T | Tortonian climatic suitability model | -4.0 | 13.2 |
| July mean temperature | Tm_07_ | °C | T | Tortonian climatic suitability model | 17.4 | 29.0 |
| Annual Mean Temperature | bio1 | °C | T | Tortonian climatic suitability model | 7.8 | 20.7 |
| Temperature Seasonality (standard deviation ×100) | bio4 | °C | T | Pliocene, Quaternary climatic suitability models | 474.0 | 8881.0 |
| Mean Temperature of Wettest Quarter | bio8 | °C | T | Pliocene, Quaternary climatic suitability models | 0.2 | 20.0 |
| Mean Temperature of Driest Quarter | bio9 | °C | T | Pliocene, Quaternary climatic suitability models | 2.0 | 28.8 |
| Mean Temperature of Warmest Quarter | bio10 | °C | T | Pliocene, Quaternary climatic suitability models | 19.4 | 28.8 |
| Mean Temperature of Coldest Quarter | bio11 | °C | T | Pliocene, Quaternary climatic suitability models | -2.8 | 13.1 |
| Annual Precipitation | bio12 | mm | P | Tortonian climatic suitability model | 307 | 1424 |
| Precipitation of Wettest Month | bio13 | mm | P | Pliocene, Quaternary climatic suitability models | 42 | 206 |
| Precipitation of Driest Month | bio14 | mm | P | Pliocene, Quaternary climatic suitability models | 0 | 51 |
| Precipitation Seasonality (Coefficient of Variation) | bio15 | mm | P | Pliocene, Quaternary climatic suitability models | 16 | 114 |
| Precipitation of Wettest Quarter | bio16 | mm | P | Pliocene, Quaternary climatic suitability models | 119 | 589 |
| Precipitation of Driest Quarter | bio17 | mm | P | Pliocene, Quaternary climatic suitability models | 0 | 161 |
| Precipitation of Warmest Quarter | bio18 | mm | P | Pliocene, Quaternary climatic suitability models | 0 | 198 |
| Precipitation of Coldest Quarter | bio19 | mm | P | Pliocene, Quaternary climatic suitability models | 78 | 478 |
| Mean annual temperature range | MATR | °C | P | Tortonian climatic suitability model | 14.9 | 22.3 |
| Thornthwaite Agrometeorological Index | TAI | mm °C-^1^ | H | Tortonian climatic suitability model | 1.62 | 9.35 |
